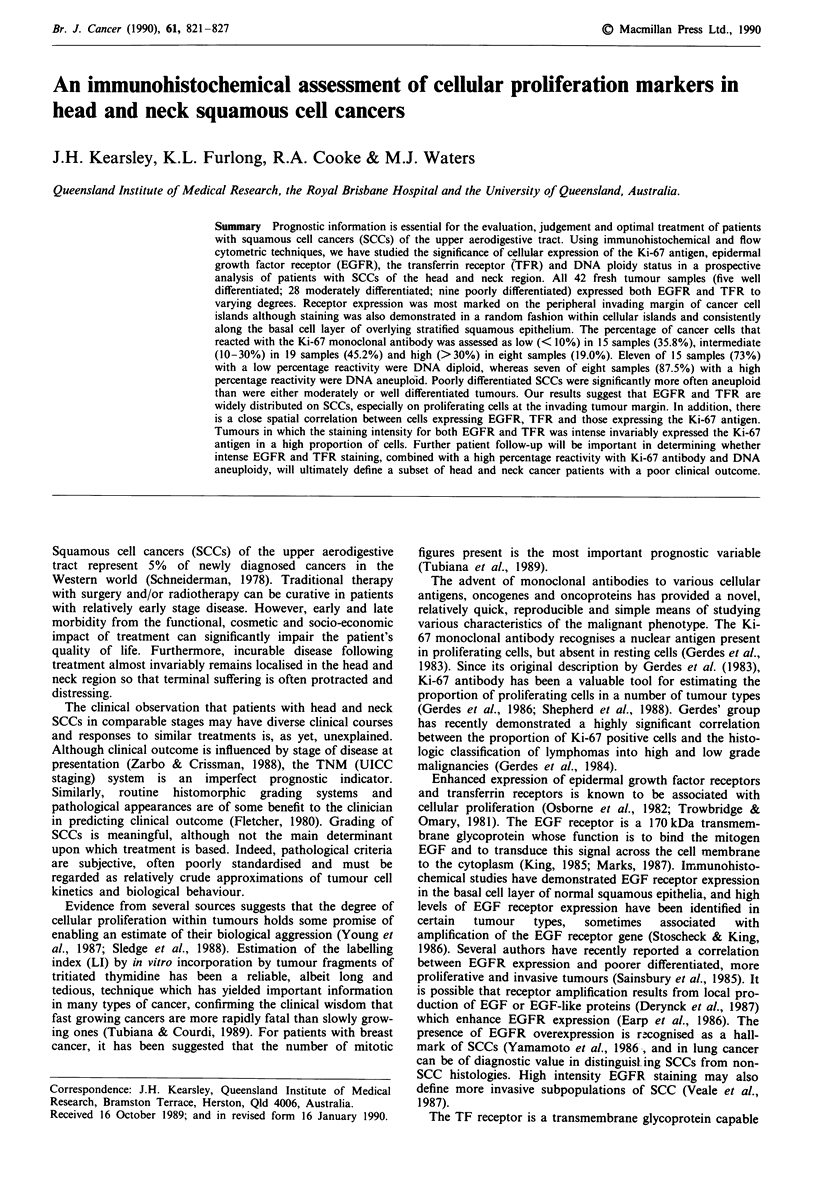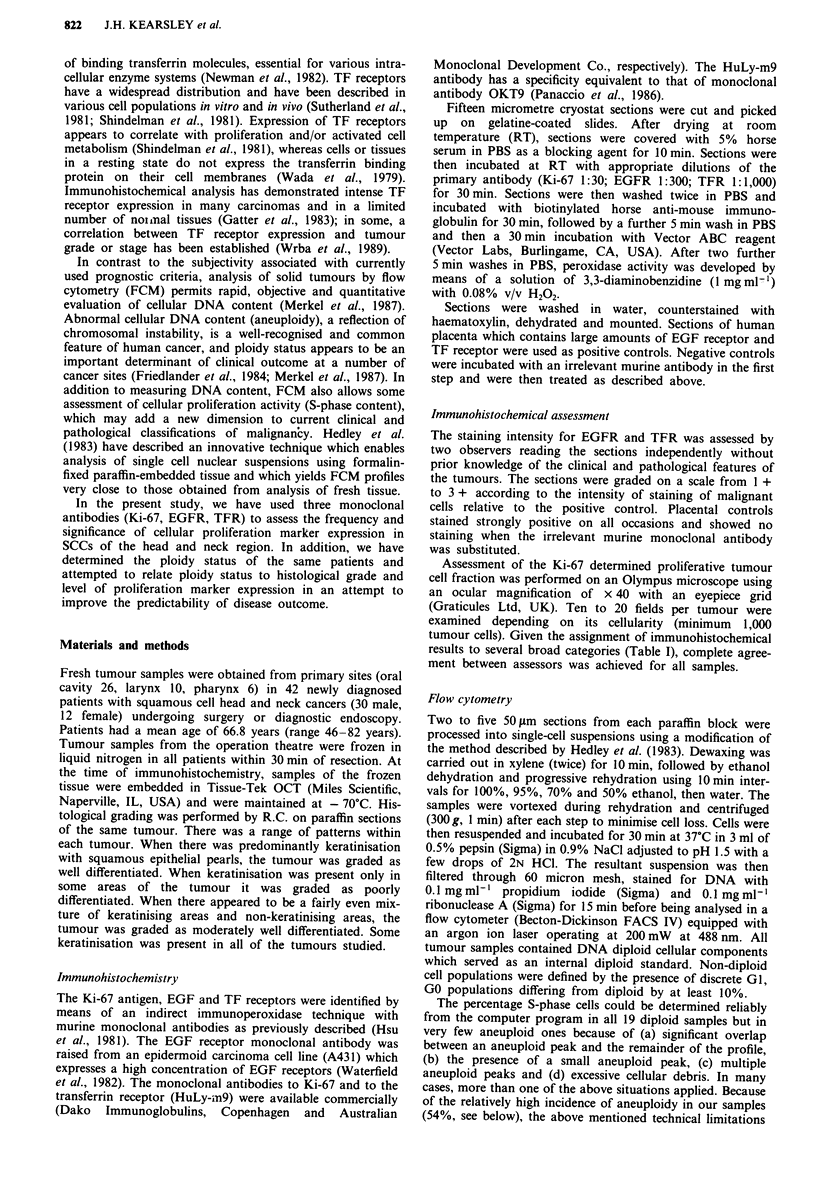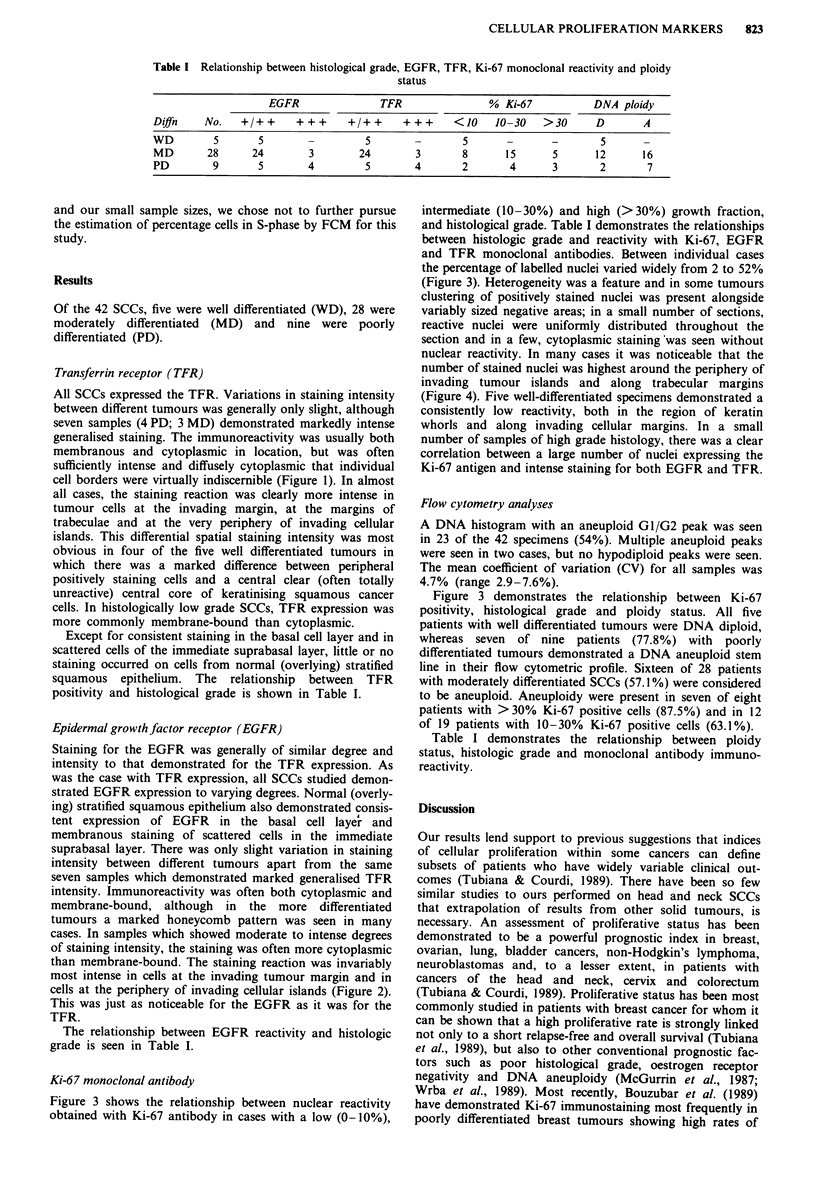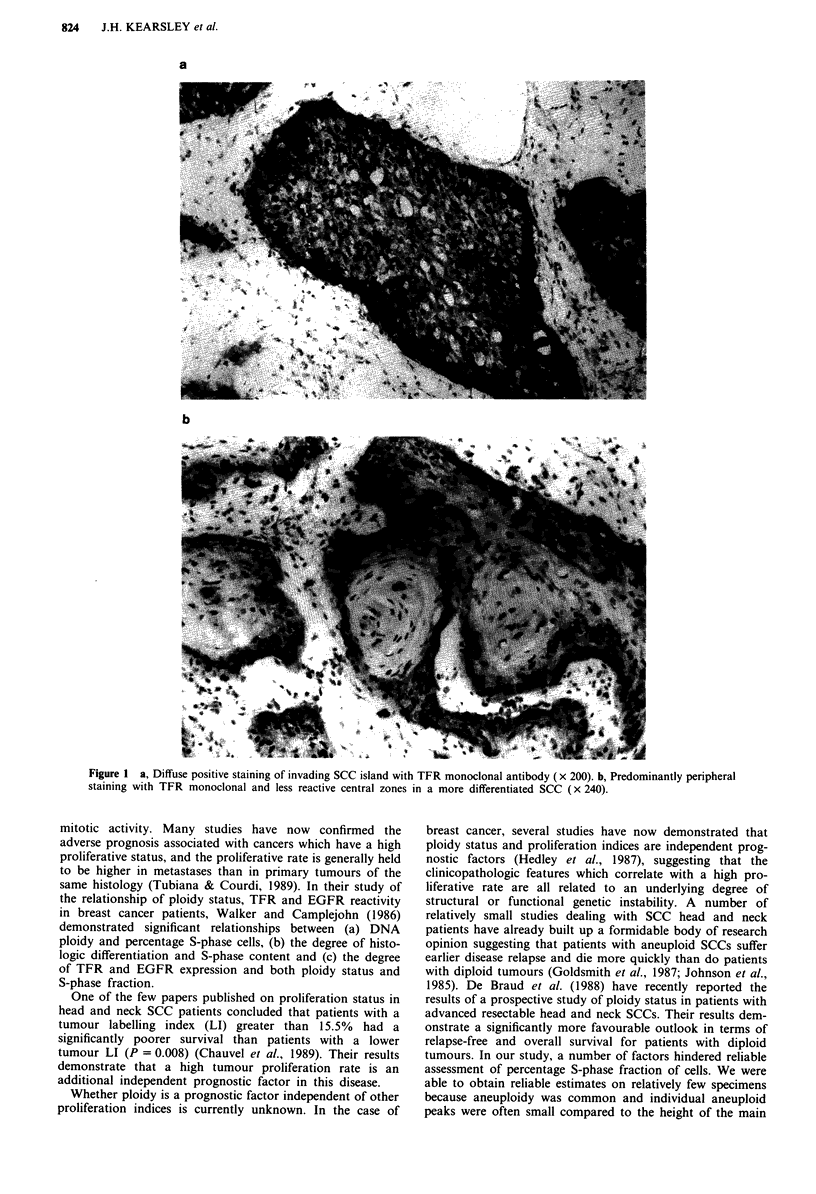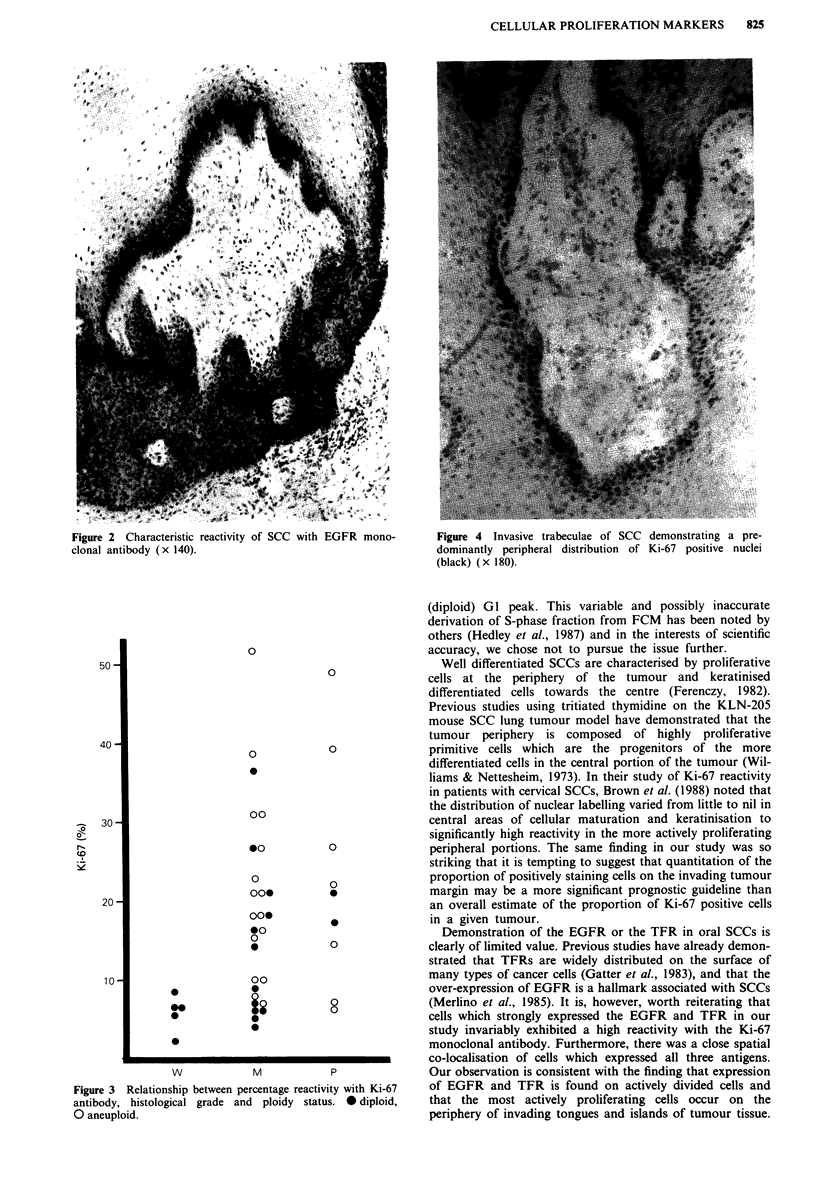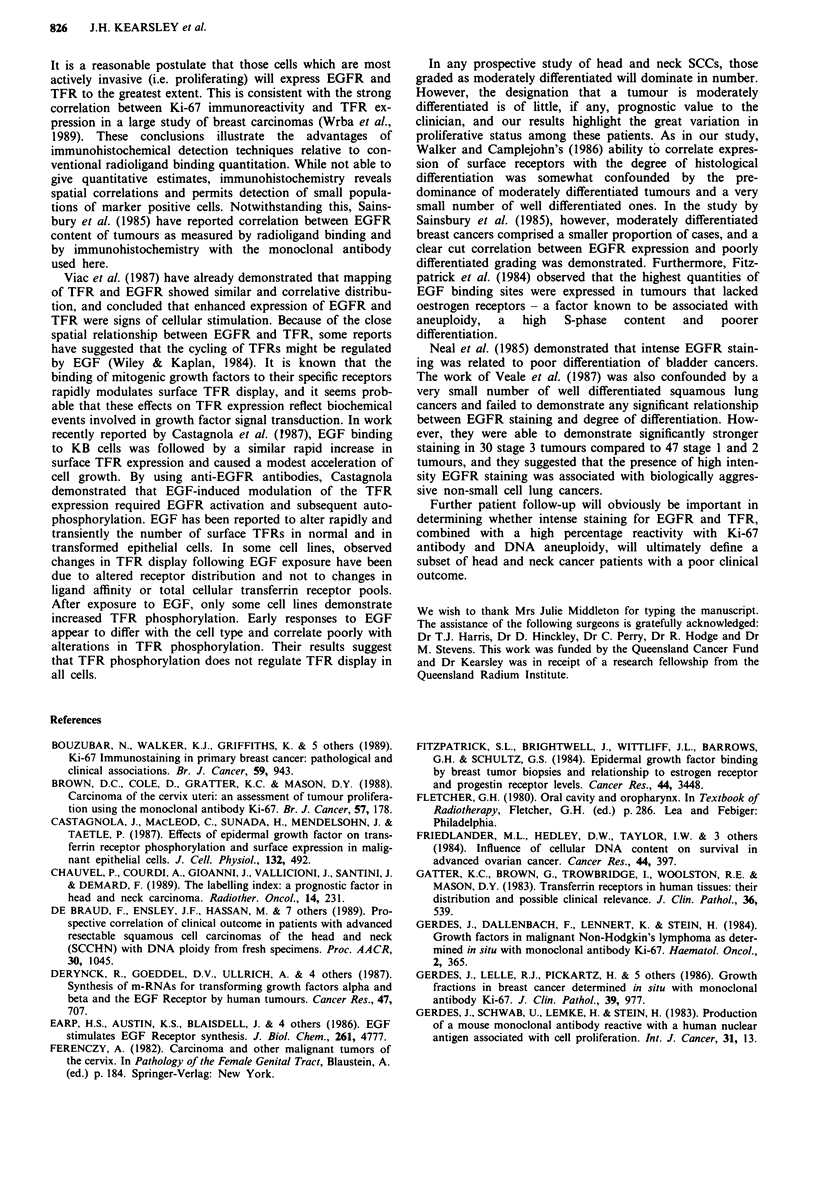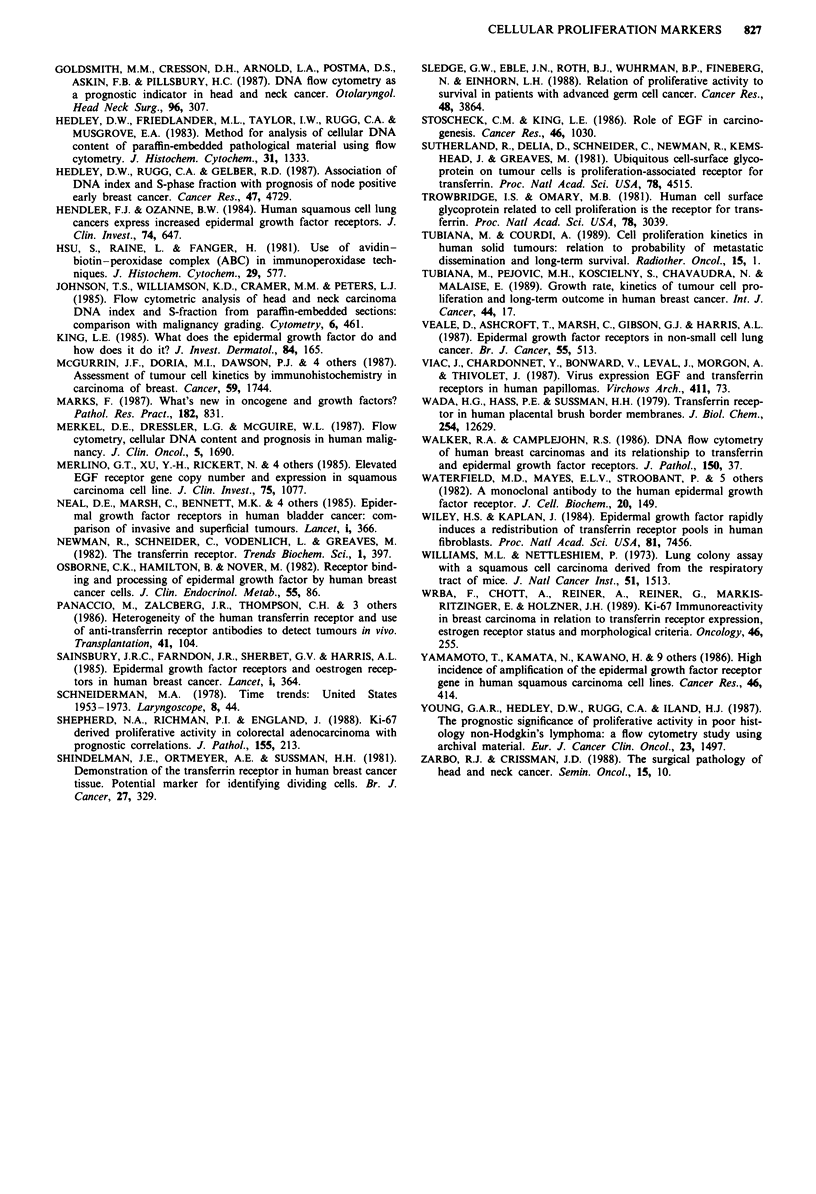# An immunohistochemical assessment of cellular proliferation markers in head and neck squamous cell cancers.

**DOI:** 10.1038/bjc.1990.184

**Published:** 1990-06

**Authors:** J. H. Kearsley, K. L. Furlong, R. A. Cooke, M. J. Waters

**Affiliations:** Queensland Institute of Medical Research, Royal Brisbane Hospital, Australia.

## Abstract

**Images:**


Br. J. Cancer (1990), 61, 821-827 © Macmillan Press Ltd., 1990

### An immunohistochemical assessment of cellular proliferation markers in head and neck squamous cell cancers

J.H. Kearsley, K.L. Furlong, R.A. Cooke & M.J. Waters

*Queensland Institute of Medical Research, the Royal Brisbane Hospital and the University of Queensland, Australia.*

**Summary** Prognostic information is essential for the evaluation, judgement and optimal treatment of patients with squamous cell cancers (SCCs) of the upper aerodigestive tract. Using immunohistochemical and flow cytometric techniques, we have studied the significance of cellular expression of the Ki-67 antigen, epidermal growth factor receptor (EGFR), the transferrin receptor (TFR) and DNA ploidy status in a prospective analysis of patients with SCCs of the head and neck region. All 42 fresh tumour samples (five well differentiated; 28 moderately differentiated; nine poorly differentiated) expressed both EGFR and TFR to varying degrees. Receptor expression was most marked on the peripheral invading margin of cancer cell islands although staining was also demonstrated in a random fashion within cellular islands and consistently along the basal cell layer of overlying stratified squamous epithelium. The percentage of cancer cells that reacted with the Ki-67 monoclonal antibody was assessed as low (< 10%) in 15 samples (35.8%), intermediate (10-30%) in 19 samples (45.2%) and high (> 30%) in eight samples (19.0%). Eleven of 15 samples (73%) with a low percentage reactivity were DNA diploid, whereas seven of eight samples (87.5%) with a high percentage reactivity were DNA aneuploïd. Poorly differentiated SCCs were significantly more often aneuploid than were either moderately or well differentiated tumours. Our results suggest that EGFR and TFR are widely distributed on SCCs, especially on proliferating cells at the invading tumour margin. In addition, there is a close spatial correlation between cells expressing EGFR, TFR and those expressing the Ki-67 antigen. Tumours in which the staining intensity for both EGFR and TFR was intense invariably expressed the Ki-67 antigen in a high proportion of cells. Further patient follow-up will be important in determining whether intense EGFR and TFR staining, combined with a high percentage reactivity with Ki-67 antibody and DNA aneuploidy, will ultimately define a subset of head and neck cancer patients with a poor clinical outcome.

Squamous cell cancers (SCCs) of the upper aerodigestive tract represent 5% of newly diagnosed cancers in the Western world (Schneiderman, 1978). Traditional therapy with surgery and/or radiotherapy can be curative in patients with relatively early stage disease. However, early and late morbidity from the functional, cosmetic and socio-economic impact of treatment can significantly impair the patient's quality of life. Furthermore, incurable disease following treatment almost invariably remains localised in the head and neck region so that terminal suffering is often protracted and distressing.

The clinical observation that patients with head and neck SCCs in comparable stages may have diverse clinical courses and responses to similar treatments is, as yet, unexplained. Although clinical outcome is influenced by stage of disease at presentation (Zarbo & Crissman, 1988), the TNM (UICC staging) system is an imperfect prognostic indicator. Similarly, routine histomorphic grading systems and pathological appearances are of some benefit to the clinician in predicting clinical outcome (Fletcher, 1980). Grading of SCCs is meaningful, although not the main determinant upon which treatment is based. Indeed, pathological criteria are subjective, often poorly standardised and must be regarded as relatively crude approximations of tumour cell kinetics and biological behaviour.

Evidence from several sources suggests that the degree of cellular proliferation within tumours holds some promise of enabling an estimate of their biological aggression (Young *et al.*, 1987; Sledge *et al.*, 1988). Estimation of the labelling index (LI) by *in vitro* incorporation by tumour fragments of tritiated thymidine has been a reliable, albeit long and tedious, technique which has yielded important information in many types of cancer, confirming the clinical wisdom that fast growing cancers are more rapidly fatal than slowly growing ones (Tubiana & Courdi, 1989). For patients with breast cancer, it has been suggested that the number of mitotic figures present is the most important prognostic variable (Tubiana *et al.*, 1989).

The advent of monoclonal antibodies to various cellular antigens, oncogenes and oncoproteins has provided a novel, relatively quick, reproducible and simple means of studying various characteristics of the malignant phenotype. The Ki-67 monoclonal antibody recognises a nuclear antigen present in proliferating cells, but absent in resting cells (Gerdes *et al.*, 1983). Since its original description by Gerdes *et al.* (1983), Ki-67 antibody has been a valuable tool for estimating the proportion of proliferating cells in a number of tumour types (Gerdes *et al.*, 1986; Shepherd *et al.*, 1988). Gerdes' group has recently demonstrated a highly significant correlation between the proportion of Ki-67 positive cells and the histologic classification of lymphomas into high and low grade malignancies (Gerdes *et al.*, 1984).

Enhanced expression of epidermal growth factor receptors and transferrin receptors is known to be associated with cellular proliferation (Osborne *et al.*, 1982; Trowbridge & Omary, 1981). The EGF receptor is a 170 kDa transmembrane glycoprotein whose function is to bind the mitogen EGF and to transduce this signal across the cell membrane to the cytoplasm (King, 1985; Marks, 1987). Immunohistochemical studies have demonstrated EGF receptor expression in the basal cell layer of normal squamous epithelia, and high levels of EGF receptor expression have been identified in certain tumour types, sometimes associated with amplification of the EGF receptor gene (Stoscheck & King, 1986). Several authors have recently reported a correlation between EGFR expression and poorer differentiated, more proliferative and invasive tumours (Sainsbury *et al.*, 1985). It is possible that receptor amplification results from local production of EGF or EGF-like proteins (Derynck *et al.*, 1987) which enhance EGFR expression (Earp *et al.*, 1986). The presence of EGFR overexpression is recognised as a hallmark of SCCs (Yamamoto *et al.*, 1986, and in lung cancer can be of diagnostic value in distinguishing SCCs from non-SCC histologies. High intensity EGFR staining may also define more invasive subpopulations of SCC (Veale *et al.*, 1987).

The TF receptor is a transmembrane glycoprotein capable

Correspondence: J.H. Kearsley, Queensland Institute of Medical Research, Bramston Terrace, Herston, Qld 4006, Australia.
Received 16 October 1989; and in revised form 16 January 1990.

of binding transferrin molecules, essential for various intracellular enzyme systems (Newman *et al.*, 1982). TF receptors have a widespread distribution and have been described in various cell populations *in vitro* and *in vivo* (Sutherland *et al.*, 1981; Shindelman *et al.*, 1981). Expression of TF receptors appears to correlate with proliferation and/or activated cell metabolism (Shindelman *et al.*, 1981), whereas cells or tissues in a resting state do not express the transferrin binding protein on their cell membranes (Wada *et al.*, 1979). Immunohistochemical analysis has demonstrated intense TF receptor expression in many carcinomas and in a limited number of normal tissues (Gatter *et al.*, 1983); in some, a correlation between TF receptor expression and tumour grade or stage has been established (Wrba *et al.*, 1989).

In contrast to the subjectivity associated with currently used prognostic criteria, analysis of solid tumours by flow cytometry (FCM) permits rapid, objective and quantitative evaluation of cellular DNA content (Merkel *et al.*, 1987). Abnormal cellular DNA content (aneuploidy), a reflection of chromosomal instability, is a well-recognised and common feature of human cancer, and ploidy status appears to be an important determinant of clinical outcome at a number of cancer sites (Friedlander *et al.*, 1984; Merkel *et al.*, 1987). In addition to measuring DNA content, FCM also allows some assessment of cellular proliferation activity (S-phase content), which may add a new dimension to current clinical and pathological classifications of malignancy. Hedley *et al.* (1983) have described an innovative technique which enables analysis of single cell nuclear suspensions using formalin-fixed paraffin-embedded tissue and which yields FCM profiles very close to those obtained from analysis of fresh tissue.

In the present study, we have used three monoclonal antibodies (Ki-67, EGFR, TFR) to assess the frequency and significance of cellular proliferation marker expression in SCCs of the head and neck region. In addition, we have determined the ploidy status of the same patients and attempted to relate ploidy status to histological grade and level of proliferation marker expression in an attempt to improve the predictability of disease outcome.

### Materials and methods

Fresh tumour samples were obtained from primary sites (oral cavity 26, larynx 10, pharynx 6) in 42 newly diagnosed patients with squamous cell head and neck cancers (30 male, 12 female) undergoing surgery or diagnostic endoscopy. Patients had a mean age of 66.8 years (range 46–82 years). Tumour samples from the operation theatre were frozen in liquid nitrogen in all patients within 30 min of resection. At the time of immunohistochemistry, samples of the frozen tissue were embedded in Tissue-Tek OCT (Miles Scientific, Naperville, IL, USA) and were maintained at − 70°C. Histological grading was performed by R.C. on paraffin sections of the same tumour. There was a range of patterns within each tumour. When there was predominantly keratinisation with squamous epithelial pearls, the tumour was graded as well differentiated. When keratinisation was present only in some areas of the tumour it was graded as poorly differentiated. When there appeared to be a fairly even mixture of keratinising areas and non-keratinising areas, the tumour was graded as moderately well differentiated. Some keratinisation was present in all of the tumours studied.

### *Immunohistochemistry*

The Ki-67 antigen, EGF and TF receptors were identified by means of an indirect immunoperoxidase technique with murine monoclonal antibodies as previously described (Hsu *et al.*, 1981). The EGF receptor monoclonal antibody was raised from an epidermoid carcinoma cell line (A431) which expresses a high concentration of EGF receptors (Waterfield *et al.*, 1982). The monoclonal antibodies to Ki-67 and to the transferrin receptor (HuLy-m9) were available commercially (Dako Immunoglobulins, Copenhagen and Australian Monoclonal Development Co., respectively). The HuLy-m9 antibody has a specificity equivalent to that of monoclonal antibody OKT9 (Panaccio *et al.*, 1986).

Fifteen micrometre cryostat sections were cut and picked up on gelatine-coated slides. After drying at room temperature (RT), sections were covered with 5% horse serum in PBS as a blocking agent for 10 min. Sections were then incubated at RT with appropriate dilutions of the primary antibody (Ki-67 1:30; EGFR 1:300; TFR 1:1,000) for 30 min. Sections were then washed twice in PBS and incubated with biotinylated horse anti-mouse immunoglobulin for 30 min, followed by a further 5 min wash in PBS and then a 30 min incubation with Vector ABC reagent (Vector Labs, Burlingame, CA, USA). After two further 5 min washes in PBS, peroxidase activity was developed by means of a solution of 3,3-diaminobenzidine (1 mg $ml^{-1}$) with 0.08% v/v $H_2O_2$.

Sections were washed in water, counterstained with haematoxylin, dehydrated and mounted. Sections of human placenta which contains large amounts of EGF receptor and TF receptor were used as positive controls. Negative controls were incubated with an irrelevant murine antibody in the first step and were then treated as described above.

### *Immunohistochemical assessment*

The staining intensity for EGFR and TFR was assessed by two observers reading the sections independently without prior knowledge of the clinical and pathological features of the tumours. The sections were graded on a scale from 1 + to 3 + according to the intensity of staining of malignant cells relative to the positive control. Placental controls stained strongly positive on all occasions and showed no staining when the irrelevant murine monoclonal antibody was substituted.

Assessment of the Ki-67 determined proliferative tumour cell fraction was performed on an Olympus microscope using an ocular magnification of × 40 with an eyepiece grid (Graticules Ltd, UK). Ten to 20 fields per tumour were examined depending on its cellularity (minimum 1,000 tumour cells). Given the assignment of immunohistochemical results to several broad categories (Table I), complete agreement between assessors was achieved for all samples.

### *Flow cytometry*

Two to five 50 μm sections from each paraffin block were processed into single-cell suspensions using a modification of the method described by Hedley *et al.* (1983). Dewaxing was carried out in xylene (twice) for 10 min, followed by ethanol dehydration and progressive rehydration using 10 min intervals for 100%, 95%, 70% and 50% ethanol, then water. The samples were vortexed during rehydration and centrifuged (300 *g*, 1 min) after each step to minimise cell loss. Cells were then resuspended and incubated for 30 min at 37°C in 3 ml of 0.5% pepsin (Sigma) in 0.9% NaCl adjusted to pH 1.5 with a few drops of 2N HCl. The resultant suspension was then filtered through 60 micron mesh, stained for DNA with 0.1 mg $ml^{-1}$ propidium iodide (Sigma) and 0.1 mg $ml^{-1}$ ribonuclease A (Sigma) for 15 min before being analysed in a flow cytometer (Becton-Dickinson FACS IV) equipped with an argon ion laser operating at 200 mW at 488 nm. All tumour samples contained DNA diploid cellular components which served as an internal diploid standard. Non-diploid cell populations were defined by the presence of discrete G1, G0 populations differing from diploid by at least 10%.

The percentage S-phase cells could be determined reliably from the computer program in all 19 diploid samples but in very few aneuploid ones because of (a) significant overlap between an aneuploid peak and the remainder of the profile, (b) the presence of a small aneuploid peak, (c) multiple aneuploid peaks and (d) excessive cellular debris. In many cases, more than one of the above situations applied. Because of the relatively high incidence of aneuploidy in our samples (54%, see below), the above mentioned technical limitations

**Table I** Relationship between histological grade, EGFR, TFR, Ki-67 monoclonal reactivity and ploidy status

| | | *EGFR* | | *TFR* | | *% Ki-67* | | | *DNA ploidy* | |
|---|---|---|---|---|---|---|---|---|---|---|
| *Diffn* | *No.* | +/++ | +++ | +/++ | +++ | <10 | 10–30 | >30 | *D* | *A* |
| WD | 5 | 5 | – | 5 | – | 5 | – | – | 5 | – |
| MD | 28 | 24 | 3 | 24 | 3 | 8 | 15 | 5 | 12 | 16 |
| PD | 9 | 5 | 4 | 5 | 4 | 2 | 4 | 3 | 2 | 7 |

and our small sample sizes, we chose not to further pursue the estimation of percentage cells in S-phase by FCM for this study.

### Results

Of the 42 SCCs, five were well differentiated (WD), 28 were moderately differentiated (MD) and nine were poorly differentiated (PD).

### *Transferrin receptor (TFR)*

All SCCs expressed the TFR. Variations in staining intensity between different tumours was generally only slight, although seven samples (4 PD; 3 MD) demonstrated markedly intense generalised staining. The immunoreactivity was usually both membranous and cytoplasmic in location, but was often sufficiently intense and diffusely cytoplasmic that individual cell borders were virtually indiscernible (Figure 1). In almost all cases, the staining reaction was clearly more intense in tumour cells at the invading margin, at the margins of trabeculae and at the very periphery of invading cellular islands. This differential spatial staining intensity was most obvious in four of the five well differentiated tumours in which there was a marked difference between peripheral positively staining cells and a central clear (often totally unreactive) central core of keratinising squamous cancer cells. In histologically low grade SCCs, TFR expression was more commonly membrane-bound than cytoplasmic.

Except for consistent staining in the basal cell layer and in scattered cells of the immediate suprabasal layer, little or no staining occurred on cells from normal (overlying) stratified squamous epithelium. The relationship between TFR positivity and histological grade is shown in Table I.

### *Epidermal growth factor receptor (EGFR)*

Staining for the EGFR was generally of similar degree and intensity to that demonstrated for the TFR expression. As was the case with TFR expression, all SCCs studied demonstrated EGFR expression to varying degrees. Normal (overlying) stratified squamous epithelium also demonstrated consistent expression of EGFR in the basal cell layer and membranous staining of scattered cells in the immediate suprabasal layer. There was only slight variation in staining intensity between different tumours apart from the same seven samples which demonstrated marked generalised TFR intensity. Immunoreactivity was often both cytoplasmic and membrane-bound, although in the more differentiated tumours a marked honeycomb pattern was seen in many cases. In samples which showed moderate to intense degrees of staining intensity, the staining was often more cytoplasmic than membrane-bound. The staining reaction was invariably most intense in cells at the invading tumour margin and in cells at the periphery of invading cellular islands (Figure 2). This was just as noticeable for the EGFR as it was for the TFR.

The relationship between EGFR reactivity and histologic grade is seen in Table I.

### *Ki-67 monoclonal antibody*

Figure 3 shows the relationship between nuclear reactivity obtained with Ki-67 antibody in cases with a low (0–10%), intermediate (10–30%) and high (>30%) growth fraction, and histological grade. Table I demonstrates the relationships between histologic grade and reactivity with Ki-67, EGFR and TFR monoclonal antibodies. Between individual cases the percentage of labelled nuclei varied widely from 2 to 52% (Figure 3). Heterogeneity was a feature and in some tumours clustering of positively stained nuclei was present alongside variably sized negative areas; in a small number of sections, reactive nuclei were uniformly distributed throughout the section and in a few, cytoplasmic staining was seen without nuclear reactivity. In many cases it was noticeable that the number of stained nuclei was highest around the periphery of invading tumour islands and along trabecular margins (Figure 4). Five well-differentiated specimens demonstrated a consistently low reactivity, both in the region of keratin whorls and along invading cellular margins. In a small number of samples of high grade histology, there was a clear correlation between a large number of nuclei expressing the Ki-67 antigen and intense staining for both EGFR and TFR.

### *Flow cytometry analyses*

A DNA histogram with an aneuploid G1/G2 peak was seen in 23 of the 42 specimens (54%). Multiple aneuploid peaks were seen in two cases, but no hypodiploid peaks were seen. The mean coefficient of variation (CV) for all samples was 4.7% (range 2.9–7.6%).

Figure 3 demonstrates the relationship between Ki-67 positivity, histological grade and ploidy status. All five patients with well differentiated tumours were DNA diploid, whereas seven of nine patients (77.8%) with poorly differentiated tumours demonstrated a DNA aneuploid stem line in their flow cytometric profile. Sixteen of 28 patients with moderately differentiated SCCs (57.1%) were considered to be aneuploid. Aneuploidy were present in seven of eight patients with >30% Ki-67 positive cells (87.5%) and in 12 of 19 patients with 10–30% Ki-67 positive cells (63.1%).

Table I demonstrates the relationship between ploidy status, histologic grade and monoclonal antibody immunoreactivity.

### Discussion

Our results lend support to previous suggestions that indices of cellular proliferation within some cancers can define subsets of patients who have widely variable clinical outcomes (Tubiana & Courdi, 1989). There have been so few similar studies to ours performed on head and neck SCCs that extrapolation of results from other solid tumours, is necessary. An assessment of proliferative status has been demonstrated to be a powerful prognostic index in breast, ovarian, lung, bladder cancers, non-Hodgkin's lymphoma, neuroblastomas and, to a lesser extent, in patients with cancers of the head and neck, cervix and colorectum (Tubiana & Courdi, 1989). Proliferative status has been most commonly studied in patients with breast cancer for whom it can be shown that a high proliferative rate is strongly linked not only to a short relapse-free and overall survival (Tubiana *et al.*, 1989), but also to other conventional prognostic factors such as poor histological grade, oestrogen receptor negativity and DNA aneuploidy (McGurrin *et al.*, 1987; Wrba *et al.*, 1989). Most recently, Bouzubar *et al.* (1989) have demonstrated Ki-67 immunostaining most frequently in poorly differentiated breast tumours showing high rates of a b

**Figure 1** **a**, Diffuse positive staining of invading SCC island with TFR monoclonal antibody (× 200). **b**, Predominantly peripheral staining with TFR monoclonal and less reactive central zones in a more differentiated SCC (× 240).

mitotic activity. Many studies have now confirmed the adverse prognosis associated with cancers which have a high proliferative status, and the proliferative rate is generally held to be higher in metastases than in primary tumours of the same histology (Tubiana & Courdi, 1989). In their study of the relationship of ploidy status, TFR and EGFR reactivity in breast cancer patients, Walker and Camplejohn (1986) demonstrated significant relationships between (a) DNA ploidy and percentage S-phase cells, (b) the degree of histologic differentiation and S-phase content and (c) the degree of TFR and EGFR expression and both ploidy status and S-phase fraction.

One of the few papers published on proliferation status in head and neck SCC patients concluded that patients with a tumour labelling index (LI) greater than 15.5% had a significantly poorer survival than patients with a lower tumour LI ($P = 0.008$) (Chauvel *et al.*, 1989). Their results demonstrate that a high tumour proliferation rate is an additional independent prognostic factor in this disease.

Whether ploidy is a prognostic factor independent of other proliferation indices is currently unknown. In the case of breast cancer, several studies have now demonstrated that ploidy status and proliferation indices are independent prognostic factors (Hedley *et al.*, 1987), suggesting that the clinicopathologic features which correlate with a high proliferative rate are all related to an underlying degree of structural or functional genetic instability. A number of relatively small studies dealing with SCC head and neck patients have already built up a formidable body of research opinion suggesting that patients with aneuploid SCCs suffer earlier disease relapse and die more quickly than do patients with diploid tumours (Goldsmith *et al.*, 1987; Johnson *et al.*, 1985). De Braud *et al.* (1988) have recently reported the results of a prospective study of ploidy status in patients with advanced resectable head and neck SCCs. Their results demonstrate a significantly more favourable outlook in terms of relapse-free and overall survival for patients with diploid tumours. In our study, a number of factors hindered reliable assessment of percentage S-phase fraction of cells. We were able to obtain reliable estimates on relatively few specimens because aneuploidy was common and individual aneuploid peaks were often small compared to the height of the main

**Figure 2** Characteristic reactivity of SCC with EGFR monoclonal antibody (× 140).

**Figure 4** Invasive trabeculae of SCC demonstrating a predominantly peripheral distribution of Ki-67 positive nuclei (black) (× 180).

**Figure 3** Relationship between percentage reactivity with Ki-67 antibody, histological grade and ploidy status. ● diploid, O aneuploid.

(diploid) G1 peak. This variable and possibly inaccurate derivation of S-phase fraction from FCM has been noted by others (Hedley *et al.*, 1987) and in the interests of scientific accuracy, we chose not to pursue the issue further.

Well differentiated SCCs are characterised by proliferative cells at the periphery of the tumour and keratinised differentiated cells towards the centre (Ferenczy, 1982). Previous studies using tritiated thymidine on the KLN-205 mouse SCC lung tumour model have demonstrated that the tumour periphery is composed of highly proliferative primitive cells which are the progenitors of the more differentiated cells in the central portion of the tumour (Williams & Nettesheim, 1973). In their study of Ki-67 reactivity in patients with cervical SCCs, Brown *et al.* (1988) noted that the distribution of nuclear labelling varied from little to nil in central areas of cellular maturation and keratinisation to significantly high reactivity in the more actively proliferating peripheral portions. The same finding in our study was so striking that it is tempting to suggest that quantitation of the proportion of positively staining cells on the invading tumour margin may be a more significant prognostic guideline than an overall estimate of the proportion of Ki-67 positive cells in a given tumour.

Demonstration of the EGFR or the TFR in oral SCCs is clearly of limited value. Previous studies have already demonstrated that TFRs are widely distributed on the surface of many types of cancer cells (Gatter *et al.*, 1983), and that the over-expression of EGFR is a hallmark associated with SCCs (Merlino *et al.*, 1985). It is, however, worth reiterating that cells which strongly expressed the EGFR and TFR in our study invariably exhibited a high reactivity with the Ki-67 monoclonal antibody. Furthermore, there was a close spatial co-localisation of cells which expressed all three antigens. Our observation is consistent with the finding that expression of EGFR and TFR is found on actively divided cells and that the most actively proliferating cells occur on the periphery of invading tongues and islands of tumour tissue.

It is a reasonable postulate that those cells which are most actively invasive (i.e. proliferating) will express EGFR and TFR to the greatest extent. This is consistent with the strong correlation between Ki-67 immunoreactivity and TFR expression in a large study of breast carcinomas (Wrba *et al.*, 1989). These conclusions illustrate the advantages of immunohistochemical detection techniques relative to conventional radioligand binding quantitation. While not able to give quantitative estimates, immunohistochemistry reveals spatial correlations and permits detection of small populations of marker positive cells. Notwithstanding this, Sainsbury *et al.* (1985) have reported correlation between EGFR content of tumours as measured by radioligand binding and by immunohistochemistry with the monoclonal antibody used here.

Viac *et al.* (1987) have already demonstrated that mapping of TFR and EGFR showed similar and correlative distribution, and concluded that enhanced expression of EGFR and TFR were signs of cellular stimulation. Because of the close spatial relationship between EGFR and TFR, some reports have suggested that the cycling of TFRs might be regulated by EGF (Wiley & Kaplan, 1984). It is known that the binding of mitogenic growth factors to their specific receptors rapidly modulates surface TFR display, and it seems probable that these effects on TFR expression reflect biochemical events involved in growth factor signal transduction. In work recently reported by Castagnola *et al.* (1987), EGF binding to KB cells was followed by a similar rapid increase in surface TFR expression and caused a modest acceleration of cell growth. By using anti-EGFR antibodies, Castagnola demonstrated that EGF-induced modulation of the TFR expression required EGFR activation and subsequent autophosphorylation. EGF has been reported to alter rapidly and transiently the number of surface TFRs in normal and in transformed epithelial cells. In some cell lines, observed changes in TFR display following EGF exposure have been due to altered receptor distribution and not to changes in ligand affinity or total cellular transferrin receptor pools. After exposure to EGF, only some cell lines demonstrate increased TFR phosphorylation. Early responses to EGF appear to differ with the cell type and correlate poorly with alterations in TFR phosphorylation. Their results suggest that TFR phosphorylation does not regulate TFR display in all cells.

In any prospective study of head and neck SCCs, those graded as moderately differentiated will dominate in number. However, the designation that a tumour is moderately differentiated is of little, if any, prognostic value to the clinician, and our results highlight the great variation in proliferative status among these patients. As in our study, Walker and Camplejohn's (1986) ability to correlate expression of surface receptors with the degree of histological differentiation was somewhat confounded by the predominance of moderately differentiated tumours and a very small number of well differentiated ones. In the study by Sainsbury *et al.* (1985), however, moderately differentiated breast cancers comprised a smaller proportion of cases, and a clear cut correlation between EGFR expression and poorly differentiated grading was demonstrated. Furthermore, Fitzpatrick *et al.* (1984) observed that the highest quantities of EGF binding sites were expressed in tumours that lacked oestrogen receptors – a factor known to be associated with aneuploidy, a high S-phase content and poorer differentiation.

Neal *et al.* (1985) demonstrated that intense EGFR staining was related to poor differentiation of bladder cancers. The work of Veale *et al.* (1987) was also confounded by a very small number of well differentiated squamous lung cancers and failed to demonstrate any significant relationship between EGFR staining and degree of differentiation. However, they were able to demonstrate significantly stronger staining in 30 stage 3 tumours compared to 47 stage 1 and 2 tumours, and they suggested that the presence of high intensity EGFR staining was associated with biologically aggressive non-small cell lung cancers.

Further patient follow-up will obviously be important in determining whether intense staining for EGFR and TFR, combined with a high percentage reactivity with Ki-67 antibody and DNA aneuploidy, will ultimately define a subset of head and neck cancer patients with a poor clinical outcome.

We wish to thank Mrs Julie Middleton for typing the manuscript. The assistance of the following surgeons is gratefully acknowledged: Dr T.J. Harris, Dr D. Hinckley, Dr C. Perry, Dr R. Hodge and Dr M. Stevens. This work was funded by the Queensland Cancer Fund and Dr Kearsley was in receipt of a research fellowship from the Queensland Radium Institute.

### References

BOUZUBAR, N., WALKER, K.J., GRIFFITHS, K. & 5 others (1989). Ki-67 Immunostaining in primary breast cancer: pathological and clinical associations. *Br. J. Cancer*, **59**, 943.

BROWN, D.C., COLE, D., GRATTER, K.C. & MASON, D.Y. (1988). Carcinoma of the cervix uteri: an assessment of tumour proliferation using the monoclonal antibody Ki-67. *Br. J. Cancer*, **57**, 178.

CASTAGNOLA, J., MACLEOD, C., SUNADA, H., MENDELSOHN, J. & TAETLE, P. (1987). Effects of epidermal growth factor on transferrin receptor phosphorylation and surface expression in malignant epithelial cells. *J. Cell. Physiol.*, **132**, 492.

CHAUVEL, P., COURDI, A., GIOANNI, J., VALLICIONI, J., SANTINI, J. & DEMARD, F. (1989). The labelling index: a prognostic factor in head and neck carcinoma. *Radiother. Oncol.*, **14**, 231.

DE BRAUD, F., ENSLEY, J.F., HASSAN, M. & 7 others (1989). Prospective correlation of clinical outcome in patients with advanced resectable squamous cell carcinomas of the head and neck (SCCHN) with DNA ploidy from fresh specimens. *Proc. AACR*, **30**, 1045.

DERYNCK, R., GOEDDEL, D.V., ULLRICH, A. & 4 others (1987). Synthesis of m-RNAs for transforming growth factors alpha and beta and the EGF Receptor by human tumours. *Cancer Res.*, **47**, 707.

EARP, H.S., AUSTIN, K.S., BLAISDELL, J. & 4 others (1986). EGF stimulates EGF Receptor synthesis. *J. Biol. Chem.*, **261**, 4777.

FERENCZY, A. (1982). Carcinoma and other malignant tumors of the cervix. In *Pathology of the Female Genital Tract*, Blaustein, A. (ed.) p. 184. Springer-Verlag: New York.

FITZPATRICK, S.L., BRIGHTWELL, J., WITTLIFF, J.L., BARROWS, G.H. & SCHULTZ, G.S. (1984). Epidermal growth factor binding by breast tumor biopsies and relationship to estrogen receptor and progestin receptor levels. *Cancer Res.*, **44**, 3448.

FLETCHER, G.H. (1980). Oral cavity and oropharynx. In *Textbook of Radiotherapy*, Fletcher, G.H. (ed.) p. 286. Lea and Febiger: Philadelphia.

FRIEDLANDER, M.L., HEDLEY, D.W., TAYLOR, I.W. & 3 others (1984). Influence of cellular DNA content on survival in advanced ovarian cancer. *Cancer Res.*, **44**, 397.

GATTER, K.C., BROWN, G., TROWBRIDGE, I., WOOLSTON, R.E. & MASON, D.Y. (1983). Transferrin receptors in human tissues: their distribution and possible clinical relevance. *J. Clin. Pathol.*, **36**, 539.

GERDES, J., DALLENBACH, F., LENNERT, K. & STEIN, H. (1984). Growth factors in malignant Non-Hodgkin's lymphoma as determined *in situ* with monoclonal antibody Ki-67. *Haematol. Oncol.*, **2**, 365.

GERDES, J., LELLE, R.J., PICKARTZ, H. & 5 others (1986). Growth fractions in breast cancer determined *in situ* with monoclonal antibody Ki-67. *J. Clin. Pathol.*, **39**, 977.

GERDES, J., SCHWAB, U., LEMKE, H. & STEIN, H. (1983). Production of a mouse monoclonal antibody reactive with a human nuclear antigen associated with cell proliferation. *Int. J. Cancer*, **31**, 13.

GOLDSMITH, M.M., CRESSON, D.H., ARNOLD, L.A., POSTMA, D.S., ASKIN, F.B. & PILLSBURY, H.C. (1987). DNA flow cytometry as a prognostic indicator in head and neck cancer. *Otolaryngol. Head Neck Surg.*, **96,** 307.

HEDLEY, D.W., FRIEDLANDER, M.L., TAYLOR, I.W., RUGG, C.A. & MUSGROVE, E.A. (1983). Method for analysis of cellular DNA content of paraffin-embedded pathological material using flow cytometry. *J. Histochem. Cytochem.*, **31,** 1333.

HEDLEY, D.W., RUGG, C.A. & GELBER, R.D. (1987). Association of DNA index and S-phase fraction with prognosis of node positive early breast cancer. *Cancer Res.*, **47,** 4729.

HENDLER, F.J. & OZANNE, B.W. (1984). Human squamous cell lung cancers express increased epidermal growth factor receptors. *J. Clin. Invest.*, **74,** 647.

HSU, S., RAINE, L. & FANGER, H. (1981). Use of avidin–biotin–peroxidase complex (ABC) in immunoperoxidase techniques. *J. Histochem. Cytochem.*, **29,** 577.

JOHNSON, T.S., WILLIAMSON, K.D., CRAMER, M.M. & PETERS, L.J. (1985). Flow cytometric analysis of head and neck carcinoma DNA index and S-fraction from paraffin-embedded sections: comparison with malignancy grading. *Cytometry*, **6,** 461.

KING, L.E. (1985). What does the epidermal growth factor do and how does it do it? *J. Invest. Dermatol.*, **84,** 165.

MCGURRIN, J.F., DORIA, M.I., DAWSON, P.J. & 4 others (1987). Assessment of tumour cell kinetics by immunohistochemistry in carcinoma of breast. *Cancer*, **59,** 1744.

MARKS, F. (1987). What's new in oncogene and growth factors? *Pathol. Res. Pract.*, **182,** 831.

MERKEL, D.E., DRESSLER, L.G. & MCGUIRE, W.L. (1987). Flow cytometry, cellular DNA content and prognosis in human malignancy. *J. Clin. Oncol.*, **5,** 1690.

MERLINO, G.T., XU, Y.-H., RICKERT, N. & 4 others (1985). Elevated EGF receptor gene copy number and expression in squamous carcinoma cell line. *J. Clin. Invest.*, **75,** 1077.

NEAL, D.E., MARSH, C., BENNETT, M.K. & 4 others (1985). Epidermal growth factor receptors in human bladder cancer: comparison of invasive and superficial tumours. *Lancet*, **i,** 366.

NEWMAN, R., SCHNEIDER, C., VODENLICH, L. & GREAVES, M. (1982). The transferrin receptor. *Trends Biochem. Sci.*, **1,** 397.

OSBORNE, C.K., HAMILTON, B. & NOVER, M. (1982). Receptor binding and processing of epidermal growth factor by human breast cancer cells. *J. Clin. Endocrinol. Metab.*, **55,** 86.

PANACCIO, M., ZALCBERG, J.R., THOMPSON, C.H. & 3 others (1986). Heterogeneity of the human transferrin receptor and use of anti-transferrin receptor antibodies to detect tumours *in vivo*. *Transplantation*, **41,** 104.

SAINSBURY, J.R.C., FARNDON, J.R., SHERBET, G.V. & HARRIS, A.L. (1985). Epidermal growth factor receptors and oestrogen receptors in human breast cancer. *Lancet*, **i,** 364.

SCHNEIDERMAN, M.A. (1978). Time trends: United States 1953–1973. *Laryngoscope*, **8,** 44.

SHEPHERD, N.A., RICHMAN, P.I. & ENGLAND, J. (1988). Ki-67 derived proliferative activity in colorectal adenocarcinoma with prognostic correlations. *J. Pathol.*, **155,** 213.

SHINDELMAN, J.E., ORTMEYER, A.E. & SUSSMAN, H.H. (1981). Demonstration of the transferrin receptor in human breast cancer tissue. Potential marker for identifying dividing cells. *Br. J. Cancer*, **27,** 329.

SLEDGE, G.W., EBLE, J.N., ROTH, B.J., WUHRMAN, B.P., FINEBERG, N. & EINHORN, L.H. (1988). Relation of proliferative activity to survival in patients with advanced germ cell cancer. *Cancer Res.*, **48,** 3864.

STOSCHECK, C.M. & KING, L.E. (1986). Role of EGF in carcinogenesis. *Cancer Res.*, **46,** 1030.

SUTHERLAND, R., DELIA, D., SCHNEIDER, C., NEWMAN, R., KEMSHEAD, J. & GREAVES, M. (1981). Ubiquitous cell-surface glycoprotein on tumour cells is proliferation-associated receptor for transferrin. *Proc. Natl Acad. Sci. USA*, **78,** 4515.

TROWBRIDGE, I.S. & OMARY, M.B. (1981). Human cell surface glycoprotein related to cell proliferation is the receptor for transferrin. *Proc. Natl Acad. Sci. USA*, **78,** 3039.

TUBIANA, M. & COURDI, A. (1989). Cell proliferation kinetics in human solid tumours: relation to probability of metastatic dissemination and long-term survival. *Radiother. Oncol.*, **15,** 1.

TUBIANA, M., PEJOVIC, M.H., KOSCIELNY, S., CHAVAUDRA, N. & MALAISE, E. (1989). Growth rate, kinetics of tumour cell proliferation and long-term outcome in human breast cancer. *Int. J. Cancer*, **44,** 17.

VEALE, D., ASHCROFT, T., MARSH, C., GIBSON, G.J. & HARRIS, A.L. (1987). Epidermal growth factor receptors in non-small cell lung cancer. *Br. J. Cancer*, **55,** 513.

VIAC, J., CHARDONNET, Y., BONWARD, V., LEVAL, J., MORGON, A. & THIVOLET, J. (1987). Virus expression EGF and transferrin receptors in human papillomas. *Virchows Arch.*, **411,** 73.

WADA, H.G., HASS, P.E. & SUSSMAN, H.H. (1979). Transferrin receptor in human placental brush border membranes. *J. Biol. Chem.*, **254,** 12629.

WALKER, R.A. & CAMPLEJOHN, R.S. (1986). DNA flow cytometry of human breast carcinomas and its relationship to transferrin and epidermal growth factor receptors. *J. Pathol.*, **150,** 37.

WATERFIELD, M.D., MAYES, E.L.V., STROOBANT, P. & 5 others (1982). A monoclonal antibody to the human epidermal growth factor receptor. *J. Cell. Biochem.*, **20,** 149.

WILEY, H.S. & KAPLAN, J. (1984). Epidermal growth factor rapidly induces a redistribution of transferrin receptor pools in human fibroblasts. *Proc. Natl Acad. Sci. USA*, **81,** 7456.

WILLIAMS, M.L. & NETTLESHIEM, P. (1973). Lung colony assay with a squamous cell carcinoma derived from the respiratory tract of mice. *J. Natl Cancer Inst.*, **51,** 1513.

WRBA, F., CHOTT, A., REINER, A., REINER, G., MARKIS-RITZINGER, E. & HOLZNER, J.H. (1989). Ki-67 Immunoreactivity in breast carcinoma in relation to transferrin receptor expression, estrogen receptor status and morphological criteria. *Oncology*, **46,** 255.

YAMAMOTO, T., KAMATA, N., KAWANO, H. & 9 others (1986). High incidence of amplification of the epidermal growth factor receptor gene in human squamous carcinoma cell lines. *Cancer Res.*, **46,** 414.

YOUNG, G.A.R., HEDLEY, D.W., RUGG, C.A. & ILAND, H.J. (1987). The prognostic significance of proliferative activity in poor histology non-Hodgkin's lymphoma: a flow cytometry study using archival material. *Eur. J. Cancer Clin. Oncol.*, **23,** 1497.

ZARBO, R.J. & CRISSMAN, J.D. (1988). The surgical pathology of head and neck cancer. *Semin. Oncol.*, **15,** 10.